# Supplementary material for: Metasurface-driven full-space structured light for three-dimensional imaging
Source: Nat Commun. 2022 Oct 10;13:5920. doi: 10.1038/s41467-022-32117-2 (PMC9550774; doi:10.1038/s41467-022-32117-2)
Supplement: Supplementary file 1 — Supplementary information [file 41467_2022_32117_MOESM1_ESM.pdf]

Supplementary Information

Metasurface-driven full-space structured light for three-dimensional imaging

Gyeongtae Kim[1,#], Yeseul Kim[1,#], Jooyeong Yun[1,#], Seong-Won Moon[1], Seokwoo Kim[1], Jaekyung Kim[1], Junkyeong Park[1], Trevon Badloe[1], Inki Kim[1,2,3*], Junsuk Rho[1,4,5,6*]

[1]Department of Mechanical Engineering, Pohang University of Science and Technology (POSTECH), Pohang 37673, Republic of Korea

[2]Department of Biophysics, Institute of Quantum Biophysics, Sungkyunkwan University, Suwon 16419, Republic of Korea

[3]Department of Intelligent Precision Healthcare Convergence, Sungkyunkwan University, Suwon 16419, Republic of Korea

[4]Department of Chemical Engineering, Pohang University of Science and Technology (POSTECH), Pohang 37673, Republic of Korea

[5]POSCO-POSTECH-RIST Convergence Research Center for Flat Optics and Metaphotonics, Pohang 37673, Republic of Korea

[6]National Institute of Nanomaterials Technology (NINT), Pohang 37673, Republic of Korea

[*]Corresponding author. E-mail: jsrho@postech.ac.kr; inki.kim@skku.edu

[#]These authors contributed equally to this work

Supplementary Note 1. The effect of pixel pitch on diffraction behavior.

P_N and P_G are the pixel pitch determined from Nyquist sampling theorem and grating equation, respectively, give criteria for the choice of pixel pitch of the single supercell. The light transmitted through the metasurface can be considered as a signal with a bandwidth of $2k_0$, where k_0 is the free-space wavenumber. If such a band-limited signal is sampled with a sampling frequency of k_N, the signals are added to the spectrum with an interval of k_N, thus k_N should be larger than $2k_0$ to perfectly reconstruct a signal of $2k_0$ bandwidth. In other words, P_N needs to be smaller than half of the free-space wavelength, resulting in P_N < 316 nm. On the other hand, high-order diffraction occurs even in a single supercell when the pixel pitch is larger than P_G, as derived from the transmission grating equation described as

$$n_t \sin\theta_m = n_i \sin\theta_i + m\frac{\lambda_0}{P}, \quad (1)$$

where n_t and n_i represent the refractive index of the transmitted and incident media, θ_m and θ_i denote the angles of the mth order diffraction and incident beams, λ_0 is the vacuum wavelength, and P is the pixel pitch. To prevent high-order diffraction in a single supercell under normal incidence, P_G should be smaller than $\frac{\lambda_0}{n_t}$, resulting in the condition P_G < 633 nm.

We simulate the effect of pixel pitch on the intensity distribution of the diffracted beams by varying the pixel pitch from 300 nm to 700 nm with an interval of 100 nm with the optimized meta-atom. The range of pixel pitch spans over P_N and P_G. Below 300 nm, the neighboring nanostructures are electromagnetically coupled, in other words the evanescent electromagnetic waves of nanostructures are not diminished before reaching the neighboring nanostructures[1]. The number of pixels of a single supercell is selected to allow comparing the intensity of diffracted beams at the same diffraction order (Supplementary Table 1). The pixel pitch can be divided into three regimes: (1) $P < P_N$, (2) $P_N < P < P_G$, and (3) $P_G < P$. The intensity, angle, and number of diffracted beams which are pre-determined from the single supercell appear when repeated periodically to form a 4 by 4 supercell array (Supplementary Figure 1). The number of supercell arrays is selected as four, due to computational limitations to simulate using the FDTD method. In the first regime, all diffracted beams propagate with a moderately uniform intensity, however, the intensity drops at large angles, which is unavoidable due to the decreased number of sampling points with a fixed sampling frequency. In the second regime, the decrease in intensity of higher-order diffraction is steeper due to the decreased resolvable

spatial frequency $\frac{1}{2P}$. In the third regime, the decrease in intensity is much steeper and it should be noted that unwanted higher order diffraction occurs at the largest diffraction orders, originating from the single supercell.

Supplementary Table 1. Simulated conditions of pixel pitch and number of pixels. To compare the intensity of diffracted beams for various pixel pitch P at the same order m, the number of pixels n is adjusted to sample signal frequency $1/\lambda$ with identical sampling frequency $1/nP$. $1/2P$ denotes the resolvable spatial frequency at the metasurface.

$\frac{1}{\lambda}$ [1/μm]	P [nm]	n	$\frac{1}{2P}$ [1/μm]	$\frac{1}{nP}$ [1/μm]	m
1.58	300	12	1.67	0.278	±5
	400	9	1.25	0.278	±5
	500	7	1	0.286	±5
	600	6	0.83	0.278	±5
	700	5	0.71	0.286	±5

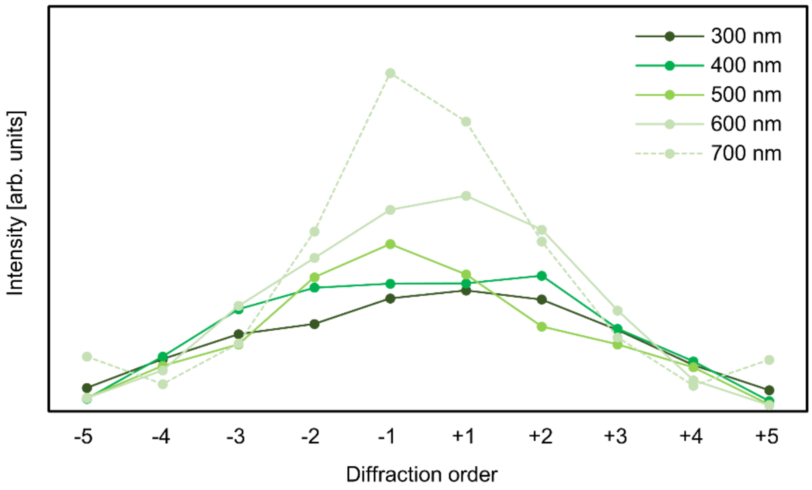

Supplementary Figure 1. The intensity distribution of diffracted beams for different pixel pitches. The larger the pixel pitch, the greater the intensity decrease in higher-order diffraction. For pixel pitch of 300 nm, which satisfies the criterion derived from both the grating equation and Nyquist sampling theorem, the intensity is the most uniform.

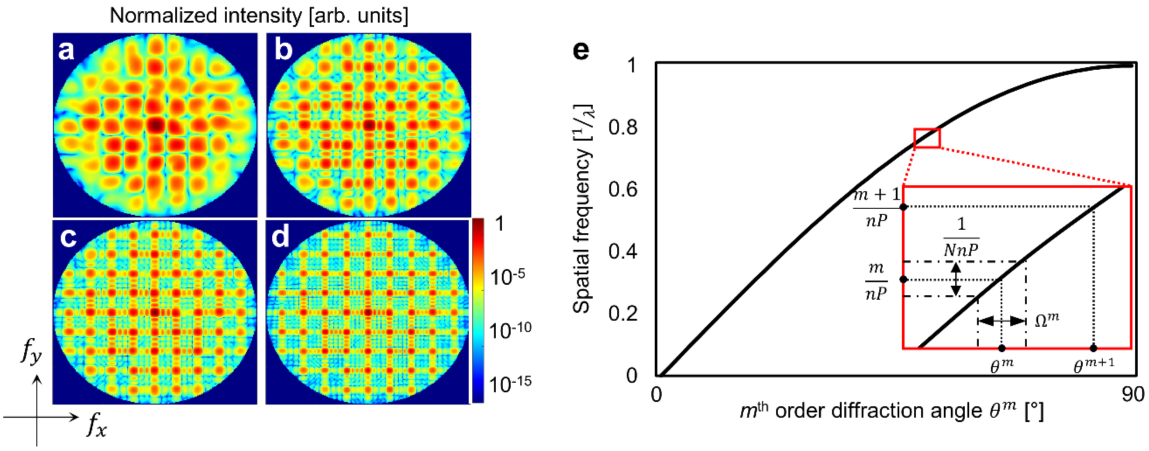

Supplementary Figure 2. The effect of the number of periodic supercells on diffracted beam diameter. a-d Simulated far-field intensity distribution in spatial frequency domain with logarithm scale under various number of supercells. The number of supercells N is varied from $N = 2$ to $N = 5$, where every supercell consists of 10 by 10 meta-atoms on a 300 nm pitch square lattice, resulting in the same number of diffracted beams. The diameter of the diffracted beams, in other words, the angle of the cone of the diffracted beams decreases as N increases. **e** The effect of sampled spatial frequency to the diffraction angle and number of supercells to angle cone of diffracted beam. Sampling period on spatial frequency $\frac{1}{nP}$, which is function of number of meta-atoms and pixel pitch, solely determines the diffraction angle. The inset shows the mth and $(m+1)^{\text{th}}$ order diffracted beams. As shown in inset, the number of supercells N is the parameter to control the angle cone of each diffracted beam, denoted as $\frac{1}{NnP}$, while not affecting the diffraction angle itself.

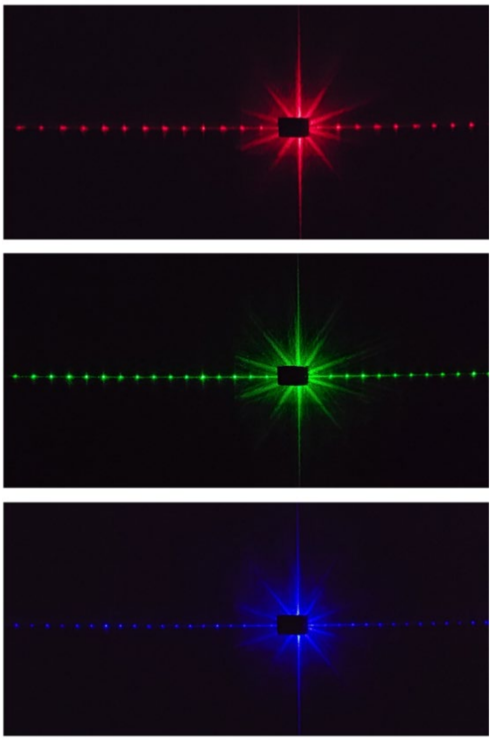

Supplementary Figure 3. Broadband operation at three primary colors and wavelength dependent diffraction of the proposed full-space diffractive metasurface. Three photographs taken under illumination of 633 nm, 532 nm, and 450 nm laser sources to the one-dimensional full-space diffractive metasurfaces. The diffraction angle become larger at the longer wavelength.

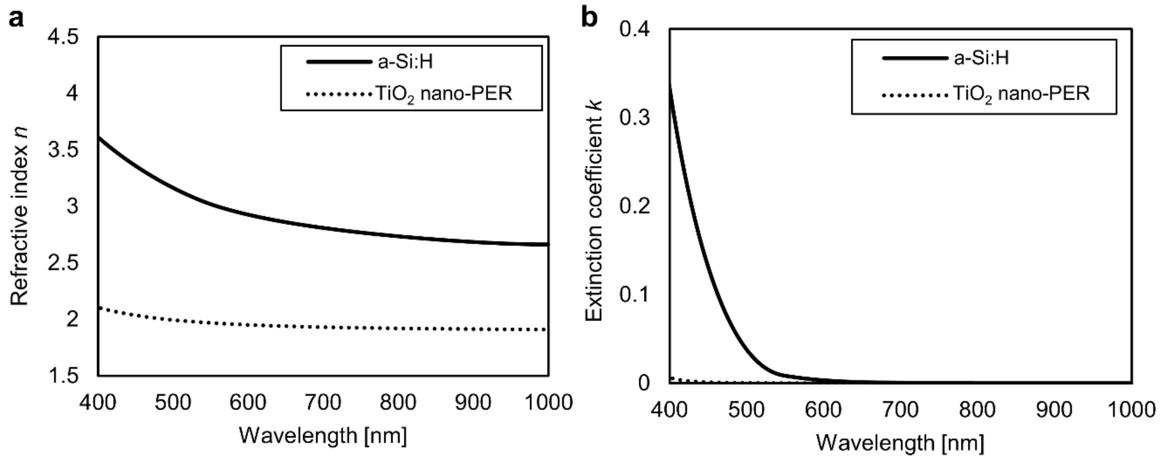

Supplementary Figure 4. Complex refractive index of a-Si:H and TiO_2 nano-PER. a Refractive index, and **b** extinction coefficient of a-Si:H and TiO_2 nano-PER. At the operation wavelength of 633 nm, both a-Si:H and TiO_2 nano-PER show high refractive index and low extinction coefficient ($n_{a-Si:H}$: 2.8, $k_{a-Si:H}$: 0.001, $n_{TiO_2\,nano-PER}$: 1.94, $k_{TiO_2\,nano-PER}$: 0.00002).

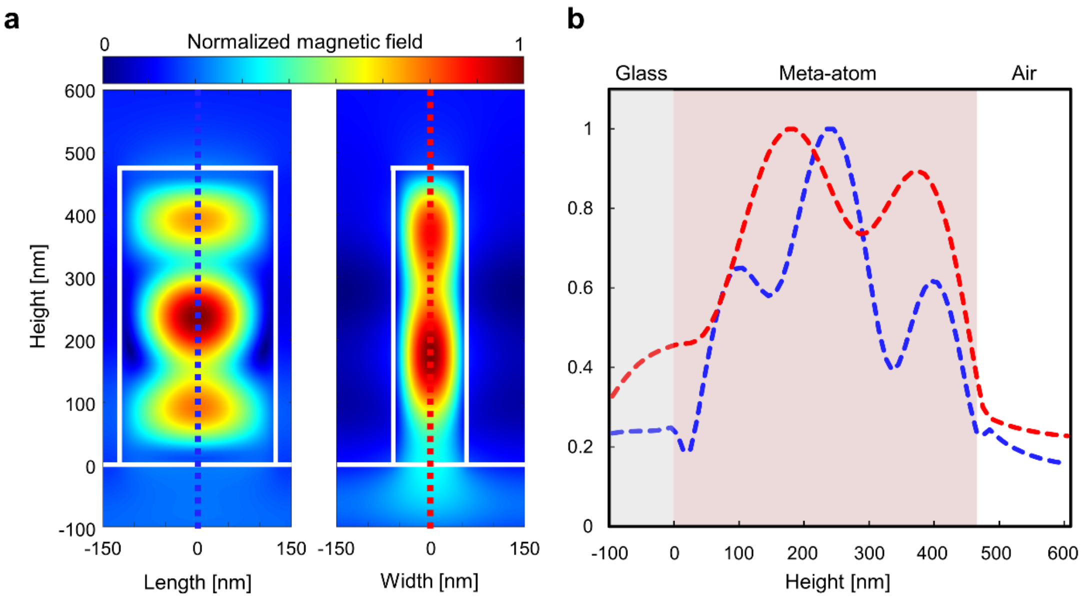

Supplementary Figure 5. Simulated magnetic field under transmitted light of linearly polarized along long and short axis of the meta-atom. a Normalized magnetic field distribution in the meta-atom (L: 250 nm, W: 110nm) under linearly polarized light incidence along length and width direction of the meta-atom. **b** Plot of field distribution along dotted line exhibiting 3.5 and 2 oscillations along long and short axis of meta-atom.

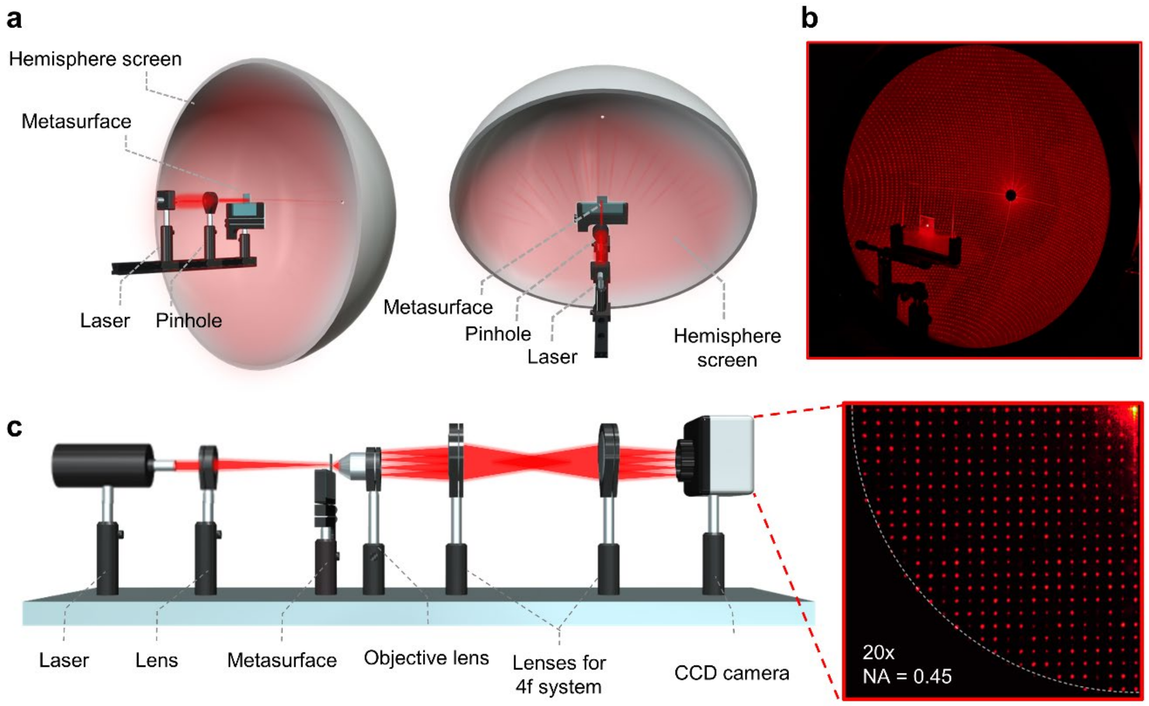

Supplementary Figure 6. Demonstration of large-angle field of view. a Schematic of the optical setup with hemisphere screen. The diameter of the hemispherical screen is 50 cm. **b** The 2D full-space structured light on hemisphere screen. **c** Fourier microscope optical setup with numerical aperture of 0.45 allowing imaging of frequency domain. The quarter of structured light is captured using CCD camera, showing diffracted beams up to 21st-order at equal intervals.

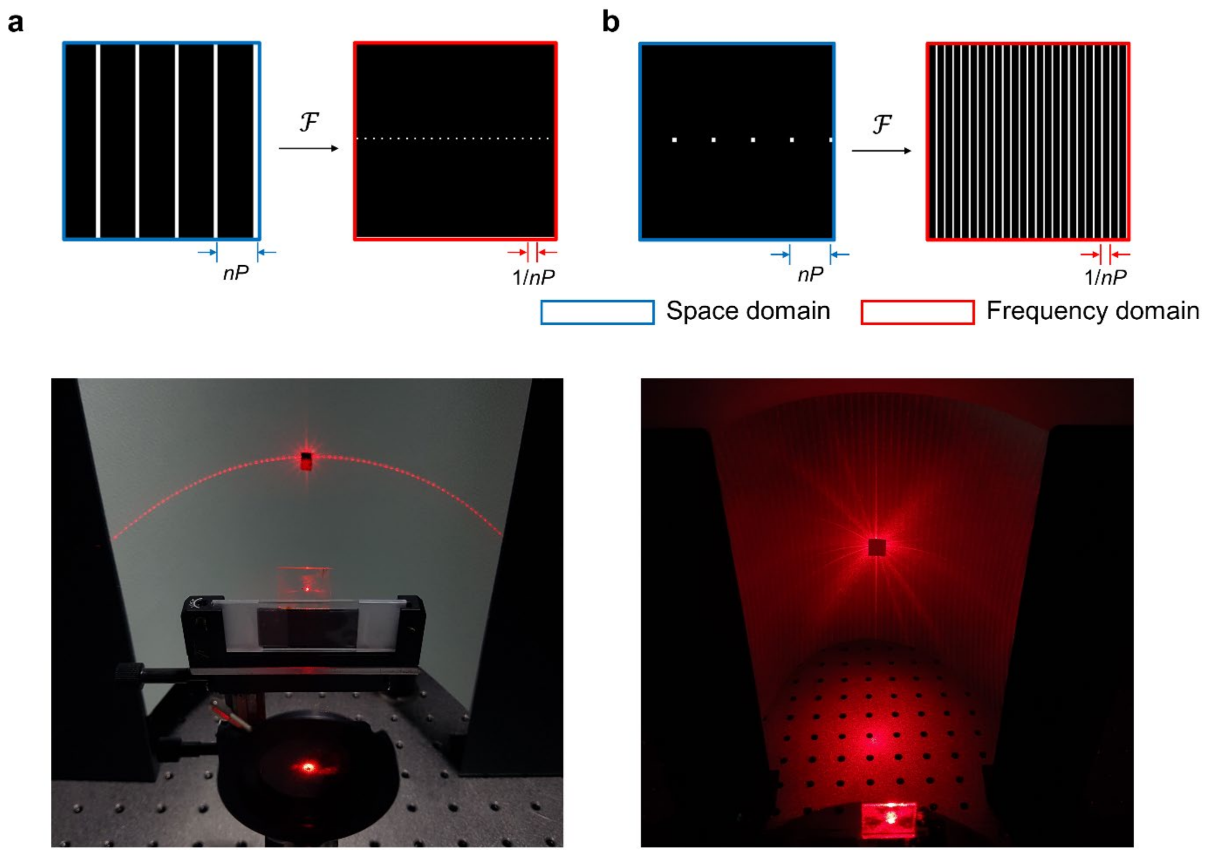

Supplementary Figure 7. Arrangement dependent structured light pattern. a Design principle of one-dimensional dot array and experimental demonstration. **b** Design principle of two-dimensional line array and experimental demonstration. Here, n and P represent the number of pixels and pixel pitch of single supercell.

Supplementary Note 2. Comparison of commercial DOE products and metasurface-based structured light projectors.

The key performance metrics of structured light projection are field of view (FOV), diffraction efficiency, zeroth-order efficiency, and spot intensity uniformity. The measured FOV of our 2D full-space diffractive metasurfaces reaches 180° with an overall diffraction efficiency of 60%. The zeroth-order makes up 32% of the total incident intensity. To measure the uniformity of the spot intensities we use a 1D full-space diffractive metasurface as there are too many dots in our 2D full-space diffractive metasurface. The root means square error (RMSE) value approaches 27.48%. The values of the key metrics are compared with previously reported metasurface-based structured light projectors[2-4] and commercial DOE products[5-8] (Supplementary Table 2). Both diffraction efficiency and spot uniformity of our metasurface are comparable to state-of-the-art DOE products and previously reported metasurface-based structured light projectors, while our 2D full-space diffractive metasurfaces exhibit a record for the FOV in the transmissive regime. It should be noted that the deflection efficiency of 46.5% is measured normalized to the transmitted light due to low transmission, originating from the bonding process of immersion lithography technology for the values in ref[3].

Supplementary Table 2. Previously reported metasurface-based structured light projectors and commercial DOE products.

**Ref.**	**Diffraction efficiency (%)**	**Spot uniformity RMSE (%)**	**Field of view (°)**	**Number of spots**
Ours	60 (2D)	27.48 (1D)	180°	6921 (2D)
[2]	55.6 (1D)<br>59.1 (2D)	39.71 (1D)*<br>38.68 (2D)*	120°	9 (1D)<br>69 (2D)
[3]	46.5 (2D)<br>(deflection efficiency)	Not reported	30°	441 (2D)
[4]	Not reported	Not reported	360°	4044 (2D)
[5]	82.2	Not reported	4.994°	144 (2D)
[6]	67	Not reported	30.77°	32761 (2D)
[7]	63	Not reported	60° (horizontal)<br>66° (vertical)	81 (2D)
[8]	Not reported	Not reported	53.3° (horizontal)<br>67.6° (vertical)	101050 (2D)

* RMSE $= \frac{1}{1/M}\sqrt{\sum_{i=1}^{M}\left(I_i - \frac{1}{M}\right)^2/M}$ where I_i is the ith order diffraction intensity normalized to the incident light intensity, and M is the total number of diffraction orders.

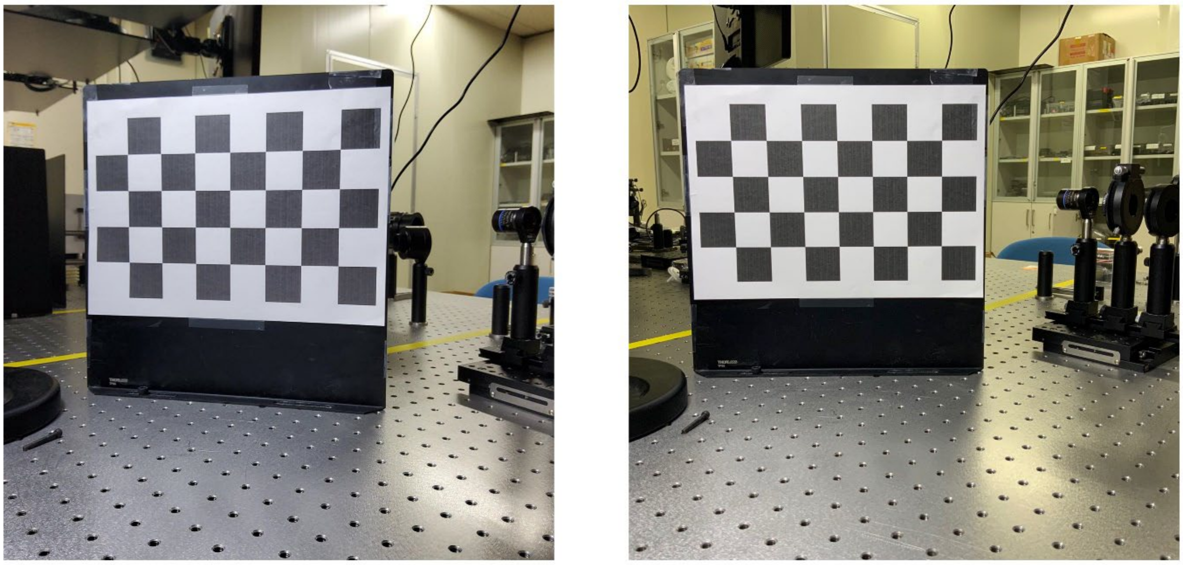

Supplementary Figure 8. Camera calibration using checkerboard pattern. The stereo cameras are calibrated using a 5x8 checkerboard pattern. 15~20 images are taken by each camera respectively, all in different angle and positions.

Supplementary Note 3. Point matching algorithm

By projecting 2D dot arrays, the geometrical information of the object scene is obtained in a 2D point cloud-form. Therefore, the correspondence problem in the stereo system can be solved by adopting various point set registration algorithms. In depth estimation experiment presented in the main text, we use coherent point drift (CPD) algorithm[9].

In CPD, the alignment of the two point sets are modeled as probability density estimation problem. One point set is represented by Gaussian mixture model (GMM) centroids, and the other point set is fitted to best match the first point set by moving coherently as a group.

Here, we denote the point set from camera 1, 2 as X, Y respectively and set Y as GMM centroids. X is considered as the data points generated by the GMM. If the total number of points in the two point sets are given as N and M, then the GMM probability function for a point $\mathbf{x}$ is expressed as,

$$p(\mathbf{x}) = \sum_{m=1}^{M+1} P(m)p(\mathbf{x}|m) \tag{2}$$

Where in $D = 2$ dimension, $p(\mathbf{x}|m)$, the Gaussian distribution centered on point $\mathbf{x}_m \in X$ with equal isotropic covariances of σ^2 is,

$$p(\mathbf{x}|m) = \frac{1}{2\pi\sigma^2} \exp^{\frac{\|\mathbf{x}-\mathbf{y}_m\|^2}{2\sigma^2}} \tag{3}$$

For all GMM components, the membership probability $P(m) = \frac{1}{\mathrm{M}}$ is equal. Denoting the weight of the uniform distribution as w $(0 \leq w \leq 1)$, the mixture model is then,

$$p(\mathbf{x}) = w\frac{1}{N} + (1-w)\sum_{m=1}^{M} \frac{1}{M} p(\mathbf{x}|m). \tag{4}$$

The GMM centroid locations are re-parameterized with a set of parameters θ, and estimated by minimizing the negative log-likelihood function with the assumption that the data is independent and identically distributed.

$$E(\theta, \sigma^2) = -\sum_{n=1}^{N} \log \sum_{m=1}^{M+1} P(m)p(\mathbf{x}|m) \tag{5}$$

Here, θ can be parameters that defines the transformation T such as rotation matrix, translation vector and scaling coefficients.

The correspondence probability between two points $\mathbf{x}_m$ and $\mathbf{y}_n$ is defined as the posterior probability of the GMM centroid given the data point:

$$P(m|\mathbf{x}_n) = \frac{P(m)p(\mathbf{x}_n|m)}{p(\mathbf{y}_n)}. \quad (6)$$

Using the expectation of maximization (EM) algorithm[10, 11], θ and σ^2 are found. Specifically, EM algorithm takes two steps. First, in the estimation step, values of θ and σ^2 are guessed and then using Bayes theorem, a posteriori probability function $P^{old}(m|\mathbf{x}_n)$ of mixture components is computed. Second, in the maximization step, the new parameter values of θ and σ^2 are found by minimizing the expectation of the complete negative log-likelihood objective function,

$$Q = -\sum_{n=1}^{N}\sum_{m=1}^{M+1} P^{old}(m|\mathbf{x}_n)\log\left(P^{new}(m)p^{new}(\mathbf{x}_n|m)\right). \quad (7)$$

The EM algorithm are iteratively conducted by alternating between the two steps until it converges.

If we ignore the constants that are independent of θ and σ^2, the above equations can be written as,

$$Q(\theta,\sigma^2) = \frac{1}{2\sigma^2}\sum_{n=1}^{N}\sum_{m=1}^{M} P^{old}(m|\mathbf{x}_n)\|\mathbf{x}_n - T(\mathbf{y}_m,\theta)\|^2 + \frac{N_P D}{2}\log\sigma^2, \quad (8)$$

where $N_P = \sum_{n=1}^{N}\sum_{m=1}^{M} P^{old}(m|\mathbf{x}_n) \leq N$ (with $N = N_p$ only if $w = 0$) and P^{old} denotes the posterior probabilities of GMM components calculated using the previous parameter values:

$$P^{old}(m|\mathbf{x}_n) = \frac{\exp\left(-\frac{1}{2}\left\|\frac{\mathbf{x}_n - T(\mathbf{y}_m,\theta^{old})}{\sigma^{old}}\right\|^2\right)}{\sum_{k=1}^{M}\exp\left(-\frac{1}{2}\left\|\frac{x_n - T(\mathbf{y}_m,\theta^{old})}{\sigma^{old}}\right\|^2\right) + c}, \quad (9)$$

where $c = (2\pi\sigma^2)^{D/2}\frac{w}{1-w}\frac{M}{N}$.

We define $T(\mathbf{y}_m;\mathbf{B},\mathbf{t}) = \mathbf{B}\mathbf{y}_m + \mathbf{t}$ as affine transformation between the two sets, where $\mathbf{B}_{D\times D}$ is an affine transformation matrix and $\mathbf{t}_{D\times 1}$ is the translation vector. Then the objective function takes the form:

$$Q(\mathbf{B},\mathbf{t},\sigma^2)=\frac{1}{2\sigma^2}\sum_{m,n=1}^{M,N}P^{old}(m|\mathbf{x}_n)\|\mathbf{x}_n-(\mathbf{B}\mathbf{y}_m+\mathbf{t})\|^2+\frac{N_pD}{2}\log\sigma^2. \quad (10)$$

Here we denote $\mathbf{P}$ as matrix whose elements are $p_{mn}=P^{old}(m|\mathbf{x}_n)$.

By directly taking the partial derivative of Q, equating them to zero and solving the resultant equations, we can solve for $\mathbf{B}$, $\mathbf{t}$, σ^2 which minimizes Q. $\mathbf{P}$ is then updated using these values. These steps are repeated until convergence and the resultant aligned point set is,

$$T(\mathbf{Y})=\mathbf{Y}\mathbf{B}^T+1\mathbf{t}^T$$

with probability correspondence given by $\mathbf{P}$. Each dot in camera 2 (set Y) is matched to a dot in camera 1 (set X) that has the highest correspondence probability among N points.

Supplementary Note 4. The depolarization effect depending on the diffraction angle.

Depolarization is a physical phenomenon in which a beam becomes depolarized under large-angle diffraction, such as when interacting with a high NA lens or wide FOV point spread metasurface because of the curvature of their spherical wavefront[12]. Therefore, under large angle conditions, the point shapes are affected by the complex polarization effect because the y and z direction electric field terms are non-zero, and therefore should be considered[13]. We obtained the intensity graphs with Gaussian fitting in the x and y directions by measuring the dots at an angle less than 30 degrees from normal and points at an angle of larger than 120 degrees with a CCD from the 2D dots metasurface. The point (Supplementary Fig. 9a) at a diffraction angle less than 30 degrees shows little depolarization effect, while the intensity width in the x-direction is almost twice as long as the intensity width in the y-direction when the diffraction angle is more than 120 degrees because of the enhanced depolarization effect (Supplementary Fig. 9b).

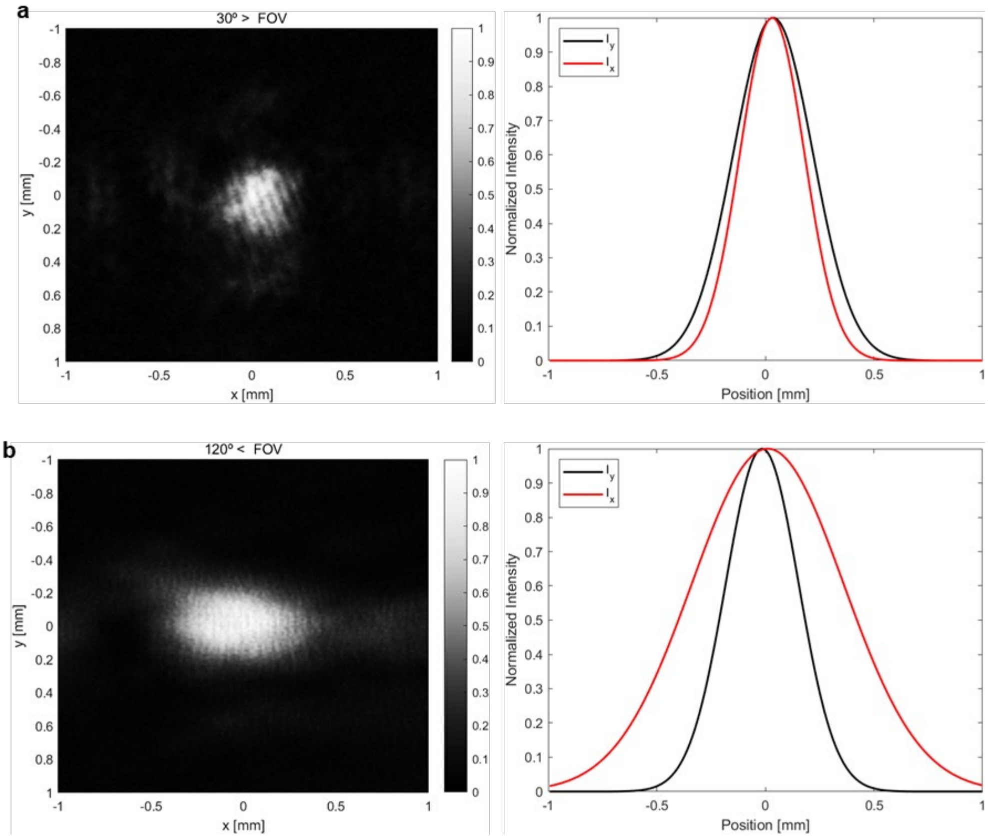

Supplementary Figure 9. The depolarization effect depending on the diffraction angle. The larger the diffraction angle, the more prominent the depolarization phenomenon appears. **a** For the point within a 30° field of view, the intensity distribution in the XY plane and the intensity plots along the x and the y directions respectively. **b** For the point outside the 120° field of view, the intensity distribution in the XY plane and the intensity plots along the x and the y directions respectively.

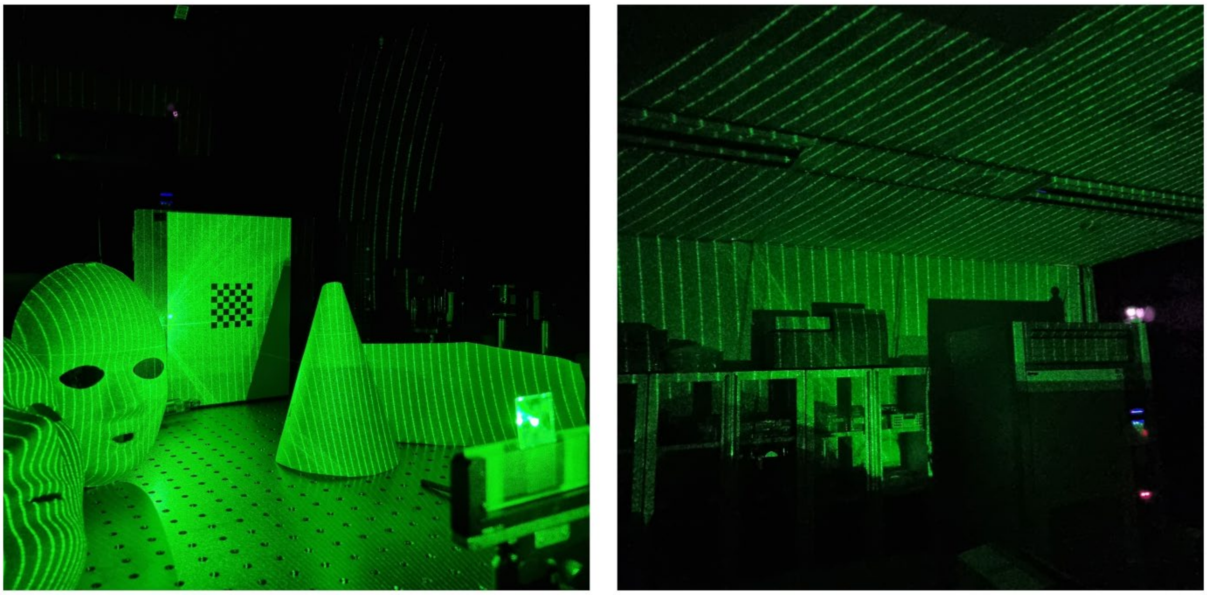

Supplementary Figure 10. Far-field operation of metasurface-based SL imaging. The power of laser used in this demonstration is 100 mW with operation wavelength of 532 nm. It demonstrates the further application of metasurface-based SL imaging for long range operation.

Supplementary References

1. Lalanne, P. & Chavel, P. Metalenses at visible wavelengths: past, present, perspectives. *Laser Photonics Rev.* **11**, 1600295 (2017).
2. Ni, Y. *et al.* Metasurface for structured light projection over 120° field of view. *Nano Lett.* **20**, 6719–6724 (2020).
3. Li, N. *et al.* Large-area pixelated metasurface beam deflector on a 12-inch glass wafer for random point generation. *Nanophotonics* **8**, 1855–1861 (2019).
4. Li, Z. *et al.* Full-space cloud of random points with a scrambling metasurface. *Light Sci. Appl.* **7**, 63 (2018).
5. Holo/Or Structured Light DOE. MS-543-905-N-A. *https://www.holoor.co.il/structured-light-doe/*
6. Holo/Or Structured Light DOE. MS-467-Q-N-X. *https://www.holoor.co.il/structured-light-doe/*
7. Holo/Or Structured Light DOE. MS-715-940-N-A. *https://www.holoor.co.il/structured-light-doe/*
8. HOLOEYE Diffractive Optical Elements. DE-R 385 *https://holoeye.com/diffractive-optics/standard-doe-plastics/*
9. Myronenko, A. & Xubo Song. Point set registration: coherent point drift. *IEEE Trans. Pattern Anal. Mach. Intell.* **32,** 2262–2275 (2010).
10. Dempster, A. P., Laird, N. M. & Rubin, D. B. Maximum likelihood from incomplete data via the EM algorithm. *J. R. Stat. Soc. B* **39,** 1-38 (1977).
11. Bishop, C. M. *Neural networks for pattern recognition.* (Oxford University Press, 1995).
12. Gu, Min. *Advanced optical imaging theory*. (Springer Science & Business Media, 2000).
13. Bahlmann, K., & Hell, S. W. Electric field depolarization in high aperture focusing with emphasis on annular apertures. *J. Microsc.* **200**, 59-67 (2000).
